# Supplementary material for: Vaccine Resistance and Hesitancy among Older Adults Who Live Alone or Only with an Older Partner in Community in the Early Stage of the Fifth Wave of COVID-19 in Hong Kong
Source: Vaccines (Basel). 2022 Jul 13;10(7):1118. doi: 10.3390/vaccines10071118 (PMC9324439; doi:10.3390/vaccines10071118)
Supplement: Supplementary file 1 [file vaccines-10-01118-s001.zip › vaccines-1787057-supplementary.pdf]

Supplement Table S1. Factors associated with vaccine uptake resistance among older people who live alone or only with an older partner in Hong Kong (N=1782)

	Multivariate regression*			
**Variables**	**aOR**	**95% CI**		**P**
**Age (years)**				
Mean (SD)	1.04	1.02	1.05	**<0.001**
**Sex**				
Male	Ref			
Female	0.85	0.68	1.06	0.158
**Living status**				
Live with an older partner	Ref			
Live alone	1.35	1.08	1.71	**0.008**
**Occupational status**				
Retirement	Ref			
Caring family/full-time/part-time/unemployed	0.76	0.44	1.32	0.332
**Social security support**				
No	Ref			
Yes (CSSA or OALA)	1.03	0.80	1.31	0.833
**Number of doctor-diagnosed chronic conditions**				
0-1	Ref			
2-3	0.99	0.78	1.26	0.961
>3	1.56	1.15	2.12	**0.005**
**Ever hospital admission over the past 6 months**				
No	Ref			
Yes	1.17	0.87	1.56	0.301
**Regular measurement of blood glucose**				
No diabetes	Ref			
Yes	1.18	0.89	1.55	0.248
Not measure regularly	1.17	0.83	1.64	0.366
**Help available when needed**				
Yes	Ref			
No	1.18	0.92	1.51	0.197
**Number of social media use in the past 2 weeks**				
0	Ref			
1	0.68	0.53	0.87	**0.003**
>1	0.45	0.32	0.64	**<0.001**
**Self-rated Health status in score**				
High (67-100)	Ref			
Middle (34-66)	1.42	1.09	1.85	**0.009**
Low (0-33)	1.57	1.21	2.05	**0.001**
**Meaning of life**				
High (5-7)	Ref			
Middle (3-4)	1.22	0.96	1.55	0.107
Low (1-2)	1.17	0.68	2.02	0.575
Missing data	1.33	0.97	1.81	0.074
**Loneliness (measured by UCLA-3)**				
0-5	Ref			
≥6	1.07	0.79	1.43	0.681
**Memory loss**				

No	Ref			
Yes, but not worry	0.87	0.69	1.10	0.251
Yes, and worry	0.99	0.71	1.39	0.970
**Depression (measured by PHQ-2)**				
0-2	Ref			
≥3	1.31	0.77	2.22	0.319
**Anxiety (measured by GAD-2)**				
0-2	Ref			
≥3	1.26	0.70	2.27	0.432

*Multivariate regression: adjusted for age, sex, living status, occupational status, social security support, number of chronic conditions, hospital admission, regular measurement of blood glucose, help available when needed, number of social medial use, self-rated health status, meaning in life, loneliness, memory loss, depression and anxiety.

Supplement Table S2 Factors associated with vaccine hesitancy among older people who live alone or only with an older partner in Hong Kong (N=1384)

	Multivariate regression*			
**Variables**	**aOR**	**95% CI**		**P**
**Age (years)**				
Mean (SD)	1.02	1.00	1.04	**0.044**
**Sex**				
Male	Ref			
Female	1.19	0.88	1.60	0.264
**Living status**				
Live with an older partner	Ref			
Live alone	1.24	0.93	1.65	0.149
**Occupational status**				
Retirement	Ref			
Caring family/full-time/part-time/unemployed	0.48	0.22	1.04	0.062
**Social security support**				
No	Ref			
Yes (CSSA or OALA)	0.95	0.70	1.30	0.766
**Number of doctor-diagnosed chronic conditions**				
0-1	Ref			
2-3	1.16	0.86	1.58	0.331
>3	1.53	1.03	2.27	**0.035**
**Ever hospital admission over the past 6 months**				
No	Ref			
Yes	1.45	1.01	2.08	**0.043**
**Regular measurement of blood glucose**				
No diabetes	Ref			
Yes	0.95	0.66	1.35	0.753
Not measure regularly	0.60	0.36	1.01	0.052
**Help available when needed**				
Yes	Ref			
No	1.00	0.72	1.38	0.977
**Number of social media use in the past 2 weeks**				
0	Ref			
1	0.72	0.52	0.99	**0.044**
>1	0.55	0.36	0.85	**0.006**
**Self-rated Health status in score**				
High (67-100)	Ref			
Middle (34-66)	1.72	1.23	2.40	**0.001**
Low (0-33)	1.51	1.06	2.13	**0.022**
**Meaning of life**				
High (5-7)	Ref			
Middle (3-4)	0.89	0.65	1.22	0.467
Low (1-2)	0.92	0.43	1.93	0.815
Missing	1.32	0.90	1.94	0.152
**Loneliness (measured by UCLA-3)**				
0-5	Ref			
≥6	1.24	0.85	1.81	0.274
**Memory loss**				

No	Ref			
Yes, but not worry	1.00	0.73	1.37	0.999
Yes, and worry	1.31	0.85	2.02	0.217
**Depression (measured by PHQ-2)**				
0-2	Ref			
≥3	0.60	0.28	1.31	0.198
**Anxiety (measured by GAD-2)**				
0-2	Ref			
≥3	1.27	0.55	2.93	0.571

*Multivariate regression: adjusted for age, sex, living status, occupational status, social security support, number of chronic conditions, hospital admission, regular measurement of blood glucose, help available when needed, number of social medial use, self-rated health status, meaning in life, loneliness, memory loss, depression and anxiety.

Supplement Table S3. Characteristics of subjects stratified by older adults who live alone or only with an older partner with different levels of meaning of life in Hong Kong

	**High (score 6-7)**		**Middle (score 4-5)**		**Low (score 1-3)**		**Missing data**		
**Variables**	N=416	%	N=1146	%	N=231	%	N=316	%	P*
**Age (years)**									
Mean (SD)	78.3	7.5	79.5	7.6	79.7	8.0	79.7	7.5	**0.035**
**Sex**									
Male	138	33.2	335	29.2	75	32.5	101	32.0	0.404
Female	278	66.8	811	70.8	156	67.5	215	68.0	
**Living status**									
Doubleton	166	39.9	418	36.5	85	36.8	106	33.5	0.360
Singleton	250	60.1	728	63.5	146	63.2	210	66.5	
**Occupational status**									
Retirement	387	93.0	1100	96.0	224	97.0	296	93.7	0.084
Caring family /Full-time/part-time/unemployed	27	6.5	46	4.0	6	2.6	13	4.1	
Missing	2	0.5	0	0.0	1	0.4	7	2.2	
**Social security support**									
No	103	24.8	257	22.4	56	24.2	72	22.8	0.772
Yes (CSSA or OALA)	313	75.2	889	77.6	175	75.8	244	77.2	
**Number of doctor-diagnosed chronic conditions**									
0-1	185	44.5	384	33.5	79	34.2	101	32.0	**<0.001**
2-3	168	40.4	511	44.6	95	41.1	130	41.1	
>3	60	14.4	249	21.7	55	23.8	83	26.3	
Missing	3	0.7	2	0.2	2	0.9	2	0.6	

**Ever hospital admission over the past 6 months**									
No	365	87.7	978	85.3	195	84.4	249	78.8	**0.014**
Yes	47	11.3	163	14.2	34	14.7	62	19.6	
Missing	4	1.0	5	0.4	2	0.9	5	1.6	
**Medical appointment in the next 6 months**									
No	97	23.3	177	15.4	51	22.1	50	15.8	**0.001**
Yes	315	75.7	965	84.2	180	77.9	261	82.6	
Missing	4	1.0	4	0.3	0	0.0	5	1.6	
**Regular measurement of blood pressure**									
No hypertension	73	17.5	110	9.6	22	9.5	21	6.6	**<0.001**
Yes	242	58.2	721	62.9	123	53.2	249	78.8	
Not measure regularly	99	23.8	307	26.8	85	36.8	42	13.3	
Missing	2	0.5	8	0.7	1	0.4	4	1.3	
**Regular measurement of blood glucose**									
No diabetes	302	72.6	760	66.3	155	67.1	235	74.4	**<0.001**
Yes	66	15.9	231	20.2	48	20.8	53	16.8	
Not measure regularly	37	8.9	139	12.1	28	12.1	12	3.8	
Missing data	11	2.6	16	1.4	0	0.0	16	5.1	
**Help available when needed**									
Yes	325	78.1	880	76.8	152	65.8	266	84.2	**<0.001**
No	91	21.9	262	22.9	79	34.2	40	12.7	
Missing	0	0.0	4	0.3	0	0.0	10	3.2	
**Number of social media use in the past 2 weeks**									
0	222	53.4	746	65.1	176	76.2	195	61.7	**<0.001**

1	113	27.2	278	24.3	36	15.6	79	25.0	
>1	81	19.5	119	10.4	19	8.2	34	10.8	
Missing data	0	0.0	3	0.3	0	0.0	8	2.5	
**Self-rated Health status in score**									
High (67-100)	220	52.9	282	24.6	42	18.2	142	44.9	**<0.001**
Middle (34-66)	126	30.3	386	33.7	56	24.2	56	17.7	
Low (0-33)	67	16.1	459	40.1	127	55.0	107	33.9	
Missing	3	0.7	19	1.7	6	2.6	11	3.5	
**Loneliness (measured by UCLA-3)**									
0-5	388	93.3	980	85.5	112	48.5	268	84.8	**<0.001**
≥6	28	6.7	165	14.4	119	51.5	43	13.6	
Missing	0	0.0	1	0.1	0	0.0	5	1.6	
**Memory loss**									
No	143	34.4	288	25.1	66	28.6	63	19.9	**<0.001**
Yes, but not worry	240	57.7	670	58.5	105	45.5	186	58.9	
Yes, and worry	33	7.9	188	16.4	60	26.0	59	18.7	
Missing	0	0.0	0	0.0	0	0.0	8	2.5	
**Depression (measured by PHQ-2)**									
0-2	409	98.3	1081	94.3	164	71.0	275	87.0	**<0.001**
≥3	5	1.2	60	5.2	66	28.6	25	7.9	
Missing	2	0.5	5	0.4	1	0.4	16	5.1	
**Anxiety (measured by GAD-2)**									
0-2	407	97.8	1093	95.4	171	74.0	286	90.5	**<0.001**
≥3	6	1.4	51	4.5	56	24.2	12	3.8	
Missing	3	0.7	2	0.2	4	1.7	18	5.7	

*P-values in the chi-square test are calculated based on available data, not including “Missing”.
